# Swimming performance, physiology, and post-activation performance enhancement following dryland transition phase warmup: A systematic review

**DOI:** 10.1371/journal.pone.0273248

**Published:** 2022-08-18

**Authors:** Max R. McKenzie, Mark R. McKean, Danielle P. Doyle, Luke W. Hogarth, Brendan J. Burkett

**Affiliations:** 1 Queensland Academy of Sport, Brisbane, Queensland, Australia; 2 School of Health and Behavioural Sciences, University of the Sunshine Coast, Sippy Downs, Queensland, Australia; 3 Swimming Australia, Brisbane, Queensland, Australia; Universidade Estadual Paulista Julio de Mesquita Filho - Campus de Bauru, BRAZIL

## Abstract

**Background:**

In swimming, the period between the end of the swimming warmup and the beginning of competition is critical to performance, here termed the transition phase. Several options are available during this phase, necessitating a systematic review to understand if optimal strategies exist.

**Objectives:**

To synthesise and critically evaluate the current literature investigating land-based warmup interventions on subsequent performance in competitive swimmers.

**Methods:**

A search of three electronic databases (PubMed, EBSCO SPORTDiscus and Web of Science) was conducted to identify original studies until February 2022. Selection criteria dictated that (i) a control condition was used, (ii) participants were ≥ 15 years of age, (iii) a pool-based warmup was done prior to the land-based warmup. A total of 25 articles met the selection criteria.

**Results:**

Reducing the transition phase duration by at least half led to consistently faster time-trial times of between 1.1–1.5% for all included studies. Passive warmups using clothing interventions resulted in mostly faster time-trial’s of 0.4–0.8% with increases in skin temperature frequent, though little change occurred in core temperature. The methodology of passive respiratory warmups were vastly different with positive time-trial’s effects ranging between 0.9–1.1% for two studies, though one reported no meaningful difference. Active warmups led to consistently faster time-trial’s between 0.7–0.9%, though the unpinning factors are not clear. Warmups which combined passive and active options frequently led to faster time-trial’s between 0.8–3%. Upper and combined limb post-activation performance enhancement led to mostly unfavourable time-trial changes. Lower limb exclusive protocols results were inconsistent, with limited beneficial effects on time-trial or start performance reported following plyometric protocols. However, there does appear merit in heavier loaded lower limb protocols.

**Conclusion:**

Each of a reduced transition phase length, and passive, active or combination warmup have demonstrated improvements in swimming performance. Conversely, PAPE protocols should be used with caution, especially when including the upper limbs.

## Introduction

The warmup prior to sporting performance is widely accepted as an essential component of training and competition. In competition, it is common for swimmers to complete a pool-based warmup before reporting to race marshals a minimum of 20 mins prior to the race [1, 2]. The period of time between the completion of the warmup and the beginning of competition has been termed the ‘transition phase’ [3]. In swimming, the transition phase is regarded as beginning once the swimmer exits the warmup pool [4]. This phase acts as the final opportunity to prepare the athlete for performance. Unlike numerous sports, the transition phase for swimming is unique as the athlete will be on land before competing in water, adding the complexity of considering how dryland interventions translate to aquatic performance.

For swimmers, the transition phase requires multiple steps including changing swimsuits, reporting to the call room at least 20 mins pre-race, and the possibility of scheduling delays, all of which can take upwards of 45 mins at major international events [1]. This length of time poses challenges when determining transition phase preparation protocols as thermal, cardiovascular, metabolic, and muscular priming outcomes may have reduced to parallel homeostasis within approximately 15 mins without specific intervention [5–7]. Therefore, interventional strategies to prolong the physiological response of the main swimming warmup, or to re-warmup, likely have the potential to enhance swimming performance at all competitive levels.

The effects of warmups are broad with improvements in physiological output, psychological readiness, and reductions in injury occurrence reported across many sports and populations [8–10]. Changes in body temperature are frequently credited as having a beneficial effect on sporting performance and skeletal muscle function due to decreased viscous resistance of blood, decreased muscle stiffness, and increased nerve conduction rates [11]. Additionally, increases in body temperature have been shown to alter anaerobic metabolism, inclusive of increased glycolysis and high-energy phosphate degradation [12]. The warmup can also have a priming effect on the cardiovascular system, enhancing subsequent intermediate work due to the elevation of baseline oxygen consumption ($\dot{V}O_{2}$) reducing the amplitude of the $\dot{V}O_{2}$ slow component [13].

Postactivation potentiation (PAP) and post-activation performance enhancement (PAPE) are warmup options which focus on maximising power output. These protocols involve the competition of a stimulus activity to enhance the neuromuscular system. Numerous studies over the past decade have demonstrated positive changes in sporting performance following PAP/PAPE intervention, particularly in sprint or highly power-derived events [14]. Both high intensity [15] and high velocity [16] stimuli have demonstrated improvements, providing a range of options for athletes. Unlike warmups protocols which focus on temperature or metabolic priming which will likely not demonstrate enhancements beyond approximately 30 to 40 mins of rest [1, 17], PAP/PAPE have demonstrated enhancements >24 hours [18].

Traditionally, warmup strategies have been divided into two categories: active or passive. Active warmup involves physical activity, often to induce a combination of thermal, cardiovascular and metabolic changes [9]. A stimulus aimed at generating a PAP or PAPE response also fits within the category of active warmup [8]. Alternatively, passive warmup does not involve physical activity, instead relying on an external source which is frequently aimed at altering body temperature [10]. Strategies including water immersion, saunas, and clothing have been investigated, with more recent publications focusing on the use of electrically heated clothing garments [4, 19]. With the introduction of heated clothing garments providing a practical method of passive warmup in many environments, a third category is now possible: combination warmup, whereby an active protocol is combined with a passive protocol [4–6, 20, 21]. With three predominant warmup interventions possible pre competition, investigation regarding the optimal intervention and timing to enhance performance is of substantial importance to practitioners.

The objective of this review was to provide guidance to practitioners on the optimal strategies by synthesing results and critically analysing studies which have investigated the effects of transitional phase warmup on subsequent swimming performance.

## Materials and methods

### Data sources

A systematic review was conducted in accordance with PRISMA (Preferred Reporting Items for Systematic reviews and Meta-Analyses) guidelines [22]. In February 2022 relevant studies were sourced from three electronic databases including PubMed, EBSCO SPORTDiscus and Web of Science. Table 1 describes the search terminology used. These terms were searched in the title and abstract of manuscripts. The reference list of each selected study was also examined for additional potentially relevant studies.

**Table 1:** Search strategy.

Variable	Search terms
Group 1: subject	Swim OR Swimmer OR Swimming
Group 2: warmup terms	“Warmup” OR "Warm up" OR Warm-up OR Transition
Group 3: outcomes	Performance OR "Time trial" OR Time-trial OR Physiology OR Post-Activation
Group 4: exclusions	Human OR Male OR Female NOT Fish NOT Rat NOT Mice

Groups combined with AND

### Selection criteria

Original peer-reviewed articles or defended thesis’ which investigated either the effects of (i) transitional phase lengths, or (ii) a land-based warmup/priming protocol on swimming performance with competitive swimming athletes with a mean age ≥15 years. Only interventional studies written in English which included a control condition were included. An in-water swim warmup must have been completed prior to experimental conditions given the current practises of swimming athletes to routinely participate in a swimming warmup prior to competition [2]. As such, studies which did not involve a pool-based warmup [15, 23–26] were excluded.

### Data extraction

Data regarding study design, population, environmental conditions, means and standard deviations of (i) performance, (ii) physiological, (iii) psychological outcomes, and reported relationships were collected by a single author. Main outcome variables included: (i) time-trial performance, (ii) core temperature pre and post performance, (iii) muscle/skin temperature pre performance, (iv) blood lactate (La-) pre and post performance, (v) heart rate (HR) pre performance, and (vi) PAP/PAPE response. Percentage change between conditions was calculated from the group mean for overall time-trial duration, accompanied by statistical significance. If the study reported percentage change, this data was used instead. These calculations were important to understand the effect of the intervention in relation to the smallest worthwhile change in elite swimming performance. Change scores and/or percentage change were also calculated for statistically significant results of all other relevant results.

### Assessment of risk of bias and study quality

Two authors independently assessed the risk of bias in each included study using the Risk of Bias 2 Tool as per the methods outlined by The Cochrane Collaboration [27]. A third author resolved the disagreement if the authors disagreed on any criteria section. Similarly, the same two authors assessed study quality using the Physiotherapy evidence database (PEDro) scale [28]. If scores differed by more than 1 point, the third author resolved the disagreement.

### Terminology and definitions

Several studies used the terms ‘core’ and ‘tympanic’ temperature interchangeably. As such, this review specifies ‘core’ temperature as measured by gastrointestinal or rectal assessment as there is conjecture regarding the validity of tympanic temperature to describe core temperature [29, 30]. Similarly, “front crawl” and “freestyle” were used interchangeably. Studies which used the front crawl technique though described the technique as “freestyle” are considered “front crawl” in this review. As PAP/PAPE is considered a subcategory of active warmup, differentiation between these and active warmup was interpreted by the authors with regards to the (i) terminology used in the study, (ii) assumed goal of thermal/cardiovascular or PAP/PAPE change, (iii) investigation of a pre and post PAP/PAPE response. Differentiation between PAP and PAPE will be described in greater detail in the discussion, though briefly, this review required a <30 s period between stimulus and performance test to be classified as PAP. If longer, it was classified as PAPE [31]. Start performance in this review encompasses dive kinetic and kinematic variables, and splits to 15 m [32].

## Results

### Study selection

**Fig 1:**
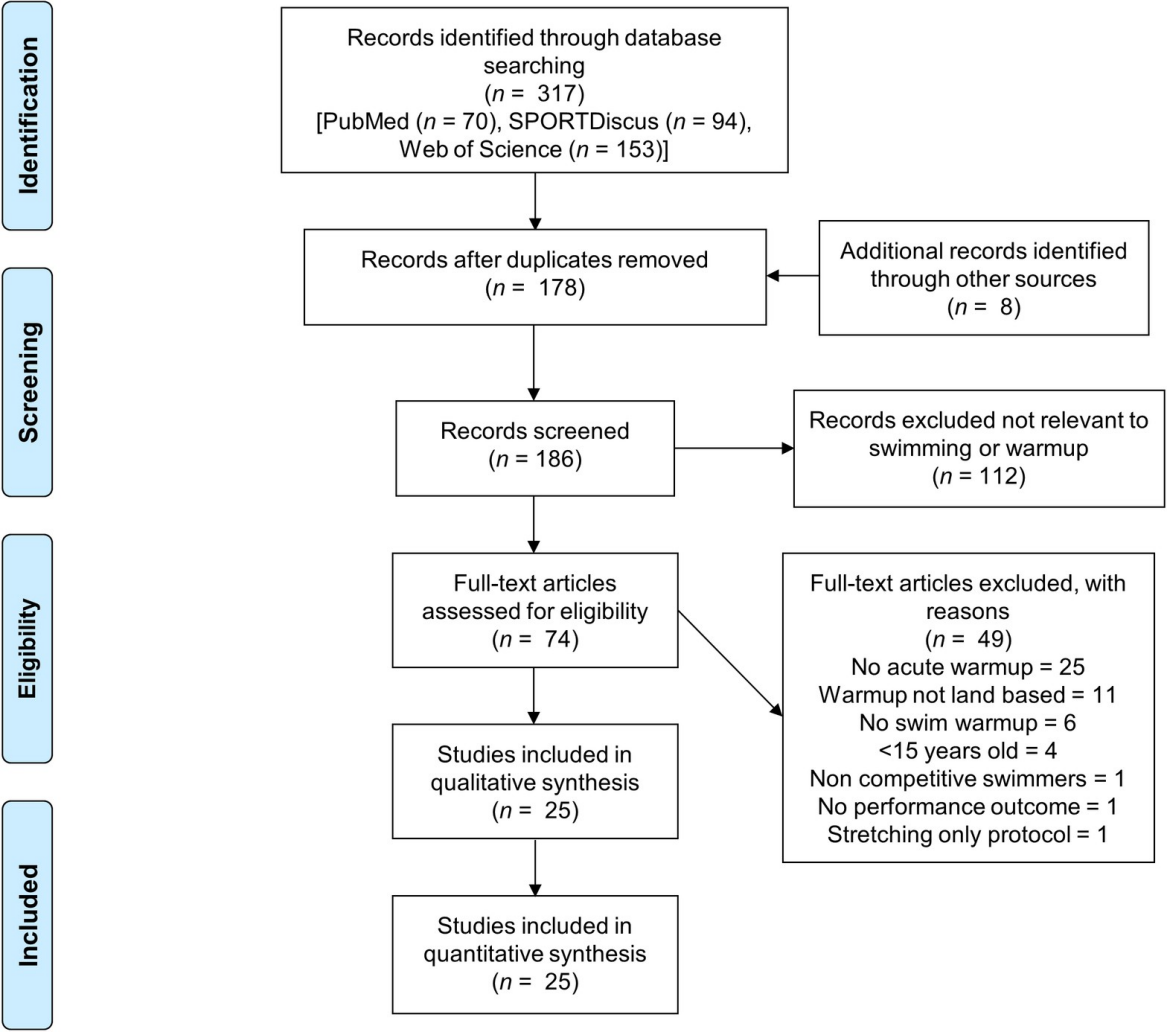
PRISMA diagram. Study selection process.

Fig 1 illustrates the schematisation of the study selection process. Initially, 317 records were identified and 25 [1, 4–7, 16, 19, 21, 33–49] studies were included in this review. These studies reported the mean and standard deviation or confidence interval for at least one primary outcome variable. A total of 41 experimental groups consisting of 334 participants were included. Tables 2–6 summarises the studies sectioned by category.

**Table 2:** Length of transition phase.

Author	Population	Transition Protocol	Performance Protocol	Effects
Zochowski et al. [48]	National (*n* = 10, 5 M age 17 ± 1.2, 5 F, age 16 ± 1.	~25 min/1500 m swim warmup. Either a 10 or 45 min transition period.	200 m main technique TT (25 m pool).	200 m TT after 10 min transition: 1.4%/1.89 s faster (*P* < 0.001); 0 to 50 m: no difference; 50 to 100 m: no difference; 100 to 150 m after 10 min transition: (%/s unknown) faster (*P* < 0.001); 150 to 200 m after 10 min transition: (%/s unknown) faster (*P* < 0.05); HR pre TT after 10 min transition: 15 BPM greater (*P* < 0.05); HR immediately post TT after 10 min transition: 4 BPM greater (*P* < 0.001); HR 3 min post TT: no difference; La- pre & post TT: no difference; RPE post TT: no difference.
West et al. [1]	International (*n* = 8, 4 M, 4 F, age 18.8 ± 1.3).	1600 m swim warmup. Either a 20 or 45 min transition period.	200 m front crawl TT (50 m pool; water temp. 28.4 ± 0.2°C, air temp. 28.2 ± 0.4° C, humidity 54 ± 1%, barometric pressure 760 ± 4 mmHg).	200 m TT after 20 mins transition: 1.5%/1.86 s faster (*P* = 0.01); 0 to 50 m after 20 mins transition: 1.6%/0.47 s faster (*P* = 0.01); 50 to 100 m after 20 mins transition: 1.7%/0.57 s faster (*P* = 0.01); 100 to 150 m after 20 mins transition: 1.5%/0.51 s faster (*P* = 0.02); 150 to 200 m after 20 mins transition: 1%/0.31 s faster (*P* = 0.17); Stroke rate: no difference; Core temp. decline after 20 mins transition: 0.4°C lower (*P* = 0.05); Core temp. pre TT after 20 mins transition: 0.3°C greater (*P* = 0.002).; Core temp. immediately post TT: no difference; Core temp. 3 min post TT: no difference.; HR pre TT, immediately post TT, & 3 min post TT: no difference.; La- pre TT: no difference; La- immediately post TT after 20 mins transition: 2.7 mmol greater (*P* = 0.001); La- 3 min post TT after 20 mins transition: 2.9 mmol greater (*P* = 0.01); RPE post TT: no difference.
Neiva et al. [42]	National (*n* = 11, 11 M, age 17.4 ± 1.8). FINA points = 534.4 ± 56.8.	1200 m swim warmup. Either 10 or 20 min transition period.	100 m front crawl TT (50 m pool; indoor, water temp. 27.6 ± 0.1°C, air temp. 27.9 ± 0.1°C, humidity 60.7 ± 0.2%).	100 m TT after 10 min transition: 1.1%/0.65 s faster (*P* < 0.01); 0 to 15 m after 10 min transition: 1.8%/0.13 s faster (*P* = 0.14); 0 to 50 m after 10 min transition: 1.5%/0.43 s faster (*P* < 0.01); 50 to 100 m after 10 min transition: 0.7%/0.22 s moderately faster (*P* = 0.08); Stroke rate 0 to 50 m after 10 mins transition: 0.02 Hz greater (*P* = 0.05); Stroke rate 50 to 100 m: no difference; Stroke length: no difference; Stroke index: no difference; Propulsion efficiency: no difference.; Core temp. decline pre TT, immediately post TT, & 15 min post TT: no difference.; Core temp. pre TT, immediately post TT, & 15 min post TT: no difference; Tympanic temp. pre TT, immediately post TT, & 15 min post TT: no difference; HR pre TT after 10 mins transition: 7 BPM greater (*P* < 0.01); HR immediately post TT after 10 mins transition: 8 BPM greater (*P* = 0.10); HR 15 mins post TT after 10 mins transition: 9% greater (*P* = 0.004); La- pre TT, immediately post TT, & 15 min post TT: no difference; $\dot{V}O_{2}$ pre TT, immediately post TT, & 15 min post TT: no difference.

Note. BPM = beats per minute; F = female; FINA = Fédération Internationale De Natation; HR = heart rate; La- = blood lactate concentration; M = male; RPE = rate of perceived exertion; TT = time-trial; $\dot{V}O_{2}$ = oxygen consumption.

**Table 3:** Passive warmup.

Author	Population	Transition Protocol	Performance Protocol	Effects
Wilson et al. [47] *‘IME combined warmup (protocol 4) vs swim only’ condition*.	International (*n* = 15, 9 M, 6 F, age 21.2 ± 1.6).	2500 m swim warmup. 2 sets of 30 inspirations at 40% of maximal inspiratory muscle pressure 1–4 mins pre TT. CON: seated rest.	100 m front crawl TT (25 m pool).	100 m TT: 1.1%/0.62 s faster (*P* = 0.05); La- pre & post TT: no difference; HR pre and post TT: no difference; PaO_2_ pre & post TT: no difference; Exhaled nitric oxide at 50 mL/s pre & post TT: no difference; Spirometry forced vital capacity pre & post TT: no difference; Maximum inspiratory & expiratory pressure pre & post TT: no difference; RPE pre & post TT: no difference; Dyspnoea scale pre & post TT: no difference.
McGowan et al. [4] *‘Passive’ condition*.	National juniors (*n* = 16, 11 M, 5 F, age 16 ± 1).	25 min/1300 m swim warmup. 30 min transition period. HEAT: heated jacket at 51°C (City heated jacket, Venture Heated Clothing). CON: tracksuit jacket. Both wore same tracksuit pants.	100 m front crawl TT (50 m pool; indoor, water temp. 27.2 ± 0.4°C, air temp. 25.8 ± 0.4°C, humidity 52.4 ± 1.3%).	100 m TT: 0.4%/0.33 s marginally faster (*P* = 0.49); 0 to 15 m: 0.2%/0.2 s possibly faster (*P* = 0.08); Turn time: 1.2%/1.23 s possibly faster (*P* = 0.05); 25 to 50 m: 0.5%/0.5 s faster (*P* = 0.00); Mid-pool velocity: no difference; Stroke efficiency: 0.2 ± 1 m^2^stroke^−1^s^−1^ greater (*P* < 0.05); Stroke length: no difference; Stroke rate: no difference; Core temp. decline: no difference; Skin temp. (iButton^®^) pre TT: 0.87°C greater (*P* = 0.04); La- pre TT: no difference; La- post TT: 1.6 mmol/L greater (*P* = 0.03); HR pre TT & post TT: no difference; RPE post TT: no difference; Core temp. & 100 m TT (all conditions pooled): *R*^2^ = 0.91 (*P* < 0.05).
Wilkins and Havenith [19]	National to international (*n* = 12; 8 M, 4 F, age = 21 ± 1.8). FINA points = 684.	30 min/1600 m swim warmup. 30 min transition period, in temp. controlled room (20.0 ± 0.2°C). HEAT: heated jacket at ~50°C (RapidFIRe Proform, Powerlet). CON: tracksuit jacket. Both wore same tracksuit pants.	50 m front crawl TT (50 m pool; indoor, water temp. 27.6 ± 0.1°C, air temp. 23.4 ± 0.1°C, humidity 55.8 ± 1.4%). 4 plyometric push-ups for M. 4 plyometric bent-knee push-ups for F.	50 m TT: 0.8%/0.21 s trending faster (*P* = 0.06); 50 m TT: M 1%/0.25 s faster (*P* < 0.05); 50 m TT: F 0.4%/0.15 s trending faster (*P* = 0.09); Time to 25 m: 1.1%/0.12 s faster (*P* < 0.05); Stroke rate 0 to 25 s: 3 stroke/min greater (*P* < 0.05); Stroke rate 25 to 50 s: 2 stroke/min greater (*P* < 0.01); Stroke count: 2 strokes greater (*P* < 0.01); SS: M 16.5% greater (*P* < 0.01); SS: F no difference; PF: M 18.1% greater (*P* < 0.01); PF: F no difference; PCP: M 16.2% greater (*P* < 0.01); PCP: F trending greater (*P* = 0.07); Tympanic temp. pre TT: no difference; Skin temp. (IR camera) pre TT: 2.3°C greater (*P* < 0.001); HR pre & post TT: no difference; RPE post TT: no difference; TS: greater from 5 to 30 mins (*P* < 0.01); TC: no difference; TC: F trend of scoring higher at 10 and 25 mins (*P* = 0.056 & *P* = 0.082).
Knight [21] *‘HEAT’ condition*.	National (*n* = 12, 6 M, 6 F, mean age 15.5 ± 2.4).	640 m swim warmup. 30 min transition period. HEAT: heated jacket (Optimum Sportswear full length coverall and Blizzard Protection System). CON: ‘normal clothing.’	100 m front crawl TT.	100 m TT: 0.4%/0.22 s faster non-significantly (*P* = 1.0); 0 to 15 m: no difference; 0 to 50 m: no difference; Tympanic temp. pre TT: no difference; Relationship tympanic temp. & 0 to 15 m: *r*_s_ = -0.641 (P = 0.025); Relationship tympanic temp. & 0 to 50 m: *r*_s_ = -0.647 (P = 0.023); Relationship tympanic temp. & 100 m TT: *r*_s_ = -0.647 (P = 0.023).
Galbraith and Willmott [41]	National (*n* = 9, 6 M, 3 F, age M: 19.5 ± 1.64, F: 21 ± 2.65). FINA points = 702.	30 min swim warmup. 30 min transition period. HEAT: warm clothing (hooded top, trousers, gloves, socks, & shoes. CON: shirt only.	100 m main technique TT (50 m pool; indoor, water temp. 27.3 ± 0.3°C, air temp. 27.9 ± 0.2°C).	100 m TT: 0.6%/0.37 s faster (*P* < 0.01); Reaction time: 2.8%/0.02 s faster (*P* < 0.05); 0 to 15 m: 1%/0.08 s faster (*P* < 0.05); 0 to 25 m: 0.7%/0.09 s faster (*P* < 0.01); First 50 m: 0.4%/0.13 s faster (*P* < 0.01); Second 50 m: 0.7%/0.24 s faster (*P* < 0.05); Tympanic temp. pre TT: 0.29°C greater (*P* < 0.01); Skin temp. (IR thermometer) pre TT: 1.55°C greater (*P* < 0.01); RPE post TT: no difference; TC pre TT: higher (*P* < 0.01).
Robertson et al. [45] *‘Warmup with apneas’ vs ‘warmup’ condition*.	Regional to national (*n* = 9, M: 9, F: 3, age 19 ± 1).	1600 m swim warmup. 20 min transition phase. PASS: 3 maximal breath holds 2 mins apart ending 2 mins pre TT. CON: seated rest.	400 m front crawl TT (25m pool; indoor, water temp.: 28.37 ± 0. 26°C, air temp. 29.13 ± 0.42°C).	400m TT: 0.1%/0.22 s faster non-significantly (*P* unknown); Stroke rate: no difference; Stroke index: no difference; HR pre & post TT: no difference; SpO_2_ pre & post TT: no difference; La- pre & post TT: no difference; Hb pre & post TT: no difference; Spleen volume pre & post TT: no difference; RPE post TT: no difference.
Ramos-Campo et al. [44] *‘Rest in hypoxia’ vs CON condition*.	National (*n* = 13, 7 M, 6 F, age 15.1 ± 2.1).	20 min/1000 m swim warmup. 30 min transition phase. PASS: 20 min resting in normobaric chamber under hypoxic conditions (FiO_2_: 15%, air temp.: 22 ± 0.5°C). CON: seated rest in normoxic conditions.	100 m front crawl TT (25 m pool; indoor, water temp. 27 ± 0.3°C, air temp. 25.6 ± 0.4°C, humidity 51.6 ± 1.2%).	100m TT: 0.9%/0.7 s faster non-significantly (*P* unknown); CMJ pre TT: no difference; Tympanic temp. pre TT: no difference; HR appx. 10 mins pre TT: no difference; HR post TT: no difference; SaO_2_ post exercise: 6.7% lower (*P* = 0.001); RPE post TT: no difference.

Note. CMJ = countermovement jump; F = female; FINA = Fédération Internationale De Natation; FiO^2^ = fraction of inspired oxygen; Hb = haemoglobin; HR = heart rate; IME = inspiratory muscle exercise; IR = infrared; La- = blood lactate concentration; M = male; PaO^2^ = partial pressure of oxygen; PCP = peak concentric power; PF = peak force; RPE = rate of perceived exertion; SpO^2^ = oxygen saturation; SS = starting strength; TC = thermal comfort; TS = thermal sensation; TT = time-trial.

**Table 4:** Active warmup.

Author	Population	Transition Protocol	Performance Protocol	Effects
McGowan et al. [4] *‘Dryland’ condition*.	National juniors (*n* = 16, 11 M, 5 F, age 16 ± 1).	25 min/1300 m swim warmup. 30 min transition period. 3x medicine ball slams (2 kg), 3x 10 s simulated underwater butterfly kicks in streamline position with BodyBlade overhead, 3x 40cm box jumps with 10 s rest between each exercise for 2 sets consecutively. Completed 16 to 21 mins prior to TT.	100 m front crawl TT (50 m pool; indoor, water temp. 27.2 ± 0.4°C, air temp. 25.8 ± 0.4°C, humidity 52.4 ± 1.3%).	100 m TT: 0.7%/0.44 s faster (*P* = 0.02); 0 to 15m: no difference; Turn time: 1.3%/1.25 s possibly faster (*P* = 0.09); 25 to 50 m: no difference; Stroke efficiency: no difference; Core temp. decline: 0.4°C possibly lower (*P* = 0.09); Core temp. pre TT: 0.2°C greater (*P* unknown); Skin temp. (iButton^®^) pre TT: no difference; La- pre & post TT: no difference; HR post exercise protocol: ~22 to 29 BPM greater (*P* = 0.00); HR pre & post TT: no difference; RPE after exercise protocol: 1.5 points greater (*P* = 0.03); RPE post TT: no difference; Core temp. & 100 m TT (all conditions pooled): *R*^2^ = 0.91 (*P* = 0.04).
Knight [21] *‘PLYO’ condition*.	National (*n* = 12, 6 M, 6 F, age 15.5 ± 2.4).	640 m swim warmup. 30 min transition period. Active: 5 x pogo, 10 m single leg alternate bounds, 5x each side single leg hops, 3x depth jumps from 30 cm. All completed for 2 sets with timing individualised.	100 m front crawl TT.	100 m TT: 0.9%/0.53 s faster (*P* = 0.029); 0 to 15 m: no difference; 0 to 50m: no difference; Tympanic temp. pre TT: no difference; Relationship tympanic temp. & 0 to 15 m: no difference; Relationship tympanic temp. & 0 to 50 m: no difference. Relationship tympanic temp. & 100 m TT: no difference.
Bagshaw [33]	National (*n* = 9, 2 M, 7 F, age 18.7 ± 4.3).	~30 min/1415 m pool warmup. 30 min transition period. Both wore same full tracksuit. Active: 40 s of jumping jacks, & 6 explosive burpees with explosive push up and squat jump for 2 sets with rest between self-selected within a total of 5 mins. Completed 5 mins prior to TT.	200 m main technique TT (air temp. 25.1 ± 0.4°C, humidity 42.6%).	200 m TT: 0.8%/1.1 s faster (*P* < 0.01); 0 to 50 m: no difference; 50 to 100m: 1.2%/0.39 s faster non-significantly (*P* unknown); 100 to 150m: 1.8%/0.64 s (*P* = 0.018); 150 to 200m: no difference; Core temp.: no difference; HR pre exercises: ~10 BPM greater (*P* < 0.05); HR post exercises: ~40 BPM greater (*P* < 0.001); HR pre TT: no difference.
Ramos-Campo et al. [44] *‘Active in normoxia’ vs CON condition*.	National (*n* = 13, 7 M, 6 F, age 15.1 ± 2.1).	20 min/1000 m swim warmup. 30 min transition phase. 20 min in normobaric chamber under normoxic conditions (FiO_2_: 20.9%, air temp.: 22 ± 0.5°C). 3x medicine ball slams (2 kg), 3x 10 s simulated underwater butterfly kicks in streamline position with BodyBlade overhead, 3x broad jumps with 10 s rest between each exercise for 2 sets consecutively. Completed 8 mins prior to TT.	100 m front crawl TT (25 m pool; indoor, water temp. 27 ± 0.3°C, air temp. 25.6 ± 0.4°C, humidity 51.6 ± 1.2%).	100m TT: 0.7%/0.5 s faster non-significantly (*P* unknown); CMJ pre TT: 1.1%/0.4 cm greater (*P* = 0.01); Tympanic temp. pre TT: 0.4°C greater (*P* unknown*)*; HR post exercise protocol: 20.6 BPM greater (*P* = 0.024); HR post TT: no difference; SaO_2_ post exercise protocol: no difference; RPE post TT: no difference.

Note. BPM = beats per minute; CMJ = countermovement jump; F = female; FiO^2^ = fraction of inspired oxygen; HR = heart rate; La- = blood lactate concentration; M = male; RPE = rate of perceived exertion; TT = time-trial.

**Table 5:** Combination warmup.

Author	Population	Transition Protocol	Performance Protocol	Effects
McGowan et al. [4] *‘Combo’ condition*.	National juniors (*n* = 16, 11 M, 5 F, age 16 ± 1).	25 min/1300 m swim warmup. 30 min transition period. HEAT: heated jacket at 51°C (City heated jacket, Venture Heated Clothing). CON: tracksuit jacket. Both wore same tracksuit pants. Active: 3x medicine ball slams (2kg), 3x 10 s simulated underwater butterfly kicks in streamline position with BodyBlade overhead, 3x 40 cm box jumps with 10 s rest between each exercise for 2 sets consecutively. Completed 16 to 21 mins prior to TT.	100 m front crawl TT (50 m pool; indoor, water temp. 27.2 ± 0.4°C, air temp. 25.8 ± 0.4°C, humidity 52.4 ± 1.3%).	100 m TT: 1.1%/0.8 s faster (*P* = 0.00); 0 to 15m: 0.4%/0.37 s faster (*P* = 0.00); Turn time: no difference; 25 to 50 m: 0.4%/0.42 s possibly faster (*P* = 0.08); Stroke efficiency: no difference; Core temp. decline: 0.51°C lower (*P* = 0.01); Core temp. pre TT: 0.48°C greater (*P* unknown); Skin temp. (iButton^®^) pre TT: 1.18°C greater (*P* = 0.03); La- pre & post TT: no difference; HR after exercise protocol: ~22 to 29 BPM greater (*P* = 0.00); HR pre & post TT: no difference; RPE post-TT: no difference; Core temp. & 100 m TT (all conditions pooled): *R*^2^ = 0.91 (*P* < 0.05).
McGowan et al. [6]	National to international (*n* = 10, 6 M, 4 F, age 20 ± 1). FINA points = 813.	25 min/1350 m swim warmup. 30 min transition period undercover. HEAT: heated pants at 51°C (Tri-Zone Heated Base Layer Bottoms, Venture Clothing). CON: tracksuit pants. Both wore same tracksuit jacket. Active: 3x medicine ball slams (2kg), 3x 10 s simulated underwater butterfly kicks in streamline position with BodyBlade overhead, 3x 4 tuck jumps with 10 s rest between each exercise for 2 sets consecutively. Completed 16 to 21 mins prior to TT.	100 m breaststroke TT (50 m pool; outdoor, water temp. 27.2 ± 0.4°C, air temp. 22.8 ± 3.8°C, humidity 62 ± 8%, wind speed 0.7 ± 0.2 m/s). 3 vertical jumps.	100 m TT: 0.3%/0.2 s faster non-significantly (*P* = 0.55); 0 to 15 m: no difference; Turn time: no difference; PP 8 min pre-TT: no difference; PP 4 min after TT for F: ~2.2% greater (*P* = 0.03); PP 4 min after TT for M: no difference; Core temp. decline: 0.19°C unclear if lower (*P* = 0.36); Skin temp. (iButton^®^) pre TT: 1.02°C greater (*P* = 0.01); Upper body surface heat pattern pre & post TT: no difference; Lower body surface heat pattern pre & post TT: no difference; La- pre & post TT: no difference; HR after exercise protocol: ~10 BPM greater (*P* = 0.02); HR pre-TT: no difference; HR post TT: 8 BMP greater (*P* = 0.02); Whole body TS: greater (*P* < 0.05); Lower body TS: greater (*P* < 0.05).
McGowan et al. [5]	National to international (*n* = 25, 12 M, 13 F, age 20 ± 3). FINA points = 807.	25 min/1350 m swim warmup. 30 min transition period. HEAT: heated jacket at 51°C (City heated jacket, Venture Heated Clothing). CON: tracksuit jacket. Both wore same tracksuit pants. Active: 3x medicine ball slams (2kg), 3x 10 s simulated underwater butterfly kicks in streamline position with BodyBlade overhead, 3x 4 tuck jumps with 10 s rest between each exercise for 2 sets consecutively. Completed 16 to 20 mins prior to TT.	100 m front crawl TT (50 m pool; outdoor, water temp. 27.2 ± 0.4°C, air temp. 22.8 ± 3.8°C, humidity 62 ± 8%, wind speed 0.8 ± 0.3 m/s). 3 CMJ’s.	100 m TT: 0.8%/0.5 s faster (*P* < 0.01); 0 to 15m: 1.5%/0.1–0.2 s faster (*P* = 0.02); Turn time: no difference; CMJ peak impulse 10 min pre TT: no difference; Core temp. decline: 0.3°C lower (*P* = 0.03); Core temp. pre TT: 0.3°C moderately greater (*P* = 0.09, ES 0.45); Skin temp (iButton^®^) relative to post-warmup.: 0.9°C greater (*P* = 0.03); Skin temp. (iButton^®^) pre TT: 1.5°C greater (*P* < 0.01); La- pre TT: no difference; La- post TT: 1.8 mmol lower (*P* = 0.03); HR after exercise protocol: ~30 BPM greater (*P* < 0.01); HR pre & post TT: no difference; tHb pre TT: 51 uM greater (*P* < 0.01); tHb difference: 1.3 uM moderately greater (*P* = 0.07); RPE after exercise protocol: 6 points greater (*P* < 0.01); RPE post TT: no difference; TC: no difference; TS whole body: greater (*P* < 0.05); TS upper body: greater (*P* < 0.05); TS lower body: greater (*P* = 0.02).
Knight [21] *‘PHEAT’ condition*.	National (*n* = 12, 6 M, 6 F, age 15.5 ± 2.4).	640 m swim warmup. 30 min transition period. HEAT: heated jacket (Optimum Sportswear full length coverall and Blizzard Protection System). CON: ‘normal clothing.’ Active: 5 x pogo, 10 m single leg alternate bounds, 5 x e.a single leg hops, 3 x depth jumps from 30 cm. All completed for 2 sets with timing individualised.	100 m front crawl TT.	100 m TT: 2%/1.15 s faster (*P* = 0.009); 0 to 15 m: 3.2%/0.21 s faster (*P* = 0.011); 0 to 50 m: no difference; Tympanic temp.: no difference; Relationship tympanic temp. & 0 to 15 m: no difference; Relationship tympanic temp. & 0 to 50 m: no difference; Relationship tympanic temp. & 100 m TT: no difference.
Ramos-Campo et al. [44] *‘Active in hypooxia’ vs CON condition*.	National (*n* = 13, 7 M, 6 F, age 15.1 ± 2.1).	20 min/1000 m swim warmup. 30 min transition phase. 20 min in normobaric chamber under hypoxic conditions (FiO_2_: 15%, air temp.: 22 ± 0.5°C). 3x medicine ball slams (2 kg), 3x 10 s simulated underwater butterfly kicks in streamline position with BodyBlade overhead, 3x broad jumps with 10 s rest between each exercise for 2 sets consecutively. Completed 8 mins prior to TT.	100 m front crawl TT (25 m pool; indoor, water temp. 27 ± 0.3°C, air temp. 25.6 ± 0.4°C, humidity 51.6 ± 1.2%).	100m TT: 3%/2.3 s faster (*P* = 0.01); CMJ: 1.1%/0.4 cm greater (*P* = 0.04); Tympanic temp.: 0.4°C greater (*P* = 0.032); HR post exercise protocol: 26.6 BPM greater (P = 0.001); HR post TT: no difference; SaO_2_ post exercise protocol: 10% lower (*P* = <0.001); RPE post TT: no difference.

Note. CMJ = countermovement jump; F = female; FINA = Fédération Internationale De Natation; FiO^2^ = fraction of inspired oxygen; HR = heart rate; La- = blood lactate concentration; M = male; RPE = rate of perceived exertion; TC = thermal comfort; tHb = local tissue total haemoglobin concentration; TS = thermal sensation; TT = time-trial.

**Table 6:** PAP/PAPE.

Author	Population	Transition Protocol	Performance Protocol	Effects
Cuenca-Fernández et al. [36] *‘Lunge’ condition*.	National (*n* = 14, 10 M, 4 F, age 17–23.	400 m swim warmup. 12 min transition phase. PAPE: 1 set of 3 reps lunge at 85% of 1RM in Smith machine completed 8 mins prior to dive testing.	Dives.	0-5m time: 2.3%/0.04 s faster (*P* = 0.03); 0–15 m time: 1.9%/0.04 s faster non-significantly (*P* unknown); Dive distance: 2.1% greater (*P* < 0.01); Flight time: 6.1% faster (*P* < 0.01); Horizontal velocity: 14.3% greater (*P* < 0.001); Angle of entry: no difference; Angle of take-off: no difference; Block time: no difference; Angular velocity of knee extension: no difference.
Cuenca-Fernández et al. [36] *‘Yoyo squat’ condition*.	National (*n* = 14, 10 M, 4 F, age 17–23.	400 m swim warmup. 12 min transition phase. PAPE: 1 set of 4 loaded reps split squats using eccentric flywheel completed 8 mins prior to dive testing.	Dives.	0-5m time: 5.88%/0.1 s faster (*P* < 0.001); 0–15 m time: 2.42%/0.18 s faster (*P* < 0.04); Dive distance: 3.4% greater (*P* < 0.01); Flight time: 15.2% faster (P < 0.001); Horizontal velocity: 34.7% greater (*P* < 0.001); Angle of entry: no difference; Angle of take-off: no difference; Block time: 6.4% faster (*P* ≤ 0.05); Angular velocity of knee extension: 18% greater (*P* < 0.005).
Sarramian et al. [46] *‘Upper-body PAPE’ condition*.	National (*n* = 18, 10 M, 8 F, age 16 ± 1.62).	PAPE: 15 swim warmup. CON: 30 min swim warmup. CON: 15 min transition period. PAPE: 3RM pull-up for 1 set. Completed at individualised optimal timing prior to TT (4, 8, or 12 mins).	50 m front crawl TT (25 m pool).	50 m TT: 1.2%/0.36 s slower (*P* = 0.046); 50 m TT M: 1.8%/0.5 s slower (*P* = 0.047); 50 m TT F: no difference.
Sarramian et al. [46] *‘Lower-body PAPE’ condition*.	National (*n* = 18, 10 M, 8 F, age 16 ± 1.62).	PAPE: 15 swim warmup. CON: 30 min swim warmup. CON: 15 min transition period. PAPE: 5 repetitions of box jumps to 41 cm with 10% of body mass using weighted vest for 1 set. Completed at individualised optimal timing prior to TT (4, 8, or 12 mins).	50 m front crawl TT (25 m pool).	50 m TT: no difference (% and *P* unknown).
Sarramian et al. [46] *‘Combined PAPE’ condition*.	National (*n* = 18, 10 M, 8 F, age 16 ± 1.62).	PAPE: 15 swim warmup. CON: 30 min swim warmup. CON: 15 min transition period. PAPE: 3RM pull-up for 1 set & 5 repetitions of box jumps to 41 cm with 10% of body mass using weighted vest for 1 set. Completed at individualised optimal timing prior to TT (4, 8, or 12 mins).	50 m front crawl TT (25 m pool).	50 m TT: no difference (% and *P* unknown).
Cuenca-Fernández et al. [38] *‘One-repetition maximum’ condition*.	National (*n* = 17, 17 M, age 18.42 ± 1.39). 74.26% of world-record.	400 m swim warmup. 10 min transition period. PAPE: 3 repetitions of shoulder extensions on 45° bench using a custom pulley & 3 repetitions of lunges both at 85% of 1RM using a Smith machine for 1 set.	50 m front crawl TT (25 m pool; indoor, water temp. 28.1°C, air temp. 29°C).	50 m TT: 0.1%/0.03 s slower non-significantly (*P* unknown); 0 to 5 m time: 3.2%/0.05 s faster (*P* < 0.05); 15 to 20 m time: 4.2%/0.12 s faster (*P* < 0.05); 5 m split times from 25 to 50 m: no difference; 10 m to 25 m & 30 m to 50 m: no difference; Block time: no difference; Dive distance: no difference; Dive time: no difference; Dive angle at take-off: 15.9% greater (*P* < 0.05); Angle of entry: no difference; Dive velocity: no difference; Underwater distance: no difference; Stroke rate at 35 m: 0.04 Hz lower (*P* < 0.05); Stroke rate at 15, 20, 45 m: no difference; Stroke length at 15 m: (% unknown) greater (*P* < 0.05); Stroke length at 20, 35, & 45 m: no difference.
Cuenca-Fernández et al. [38] *‘Eccentric flywheel’ condition*.	Competitive (*n* = 17, 17 M, age 18.42 ± 1.39). 74.26% of world-record.	400 m swim warmup. 6 min transition period. PAPE: 5 of shoulder extensions on 45° bench using a custom pulley & 4 loaded repetitions of lunges using an eccentric flywheel at the maximal voluntary contraction.	50 m front crawl TT (25 m pool; indoor, water temp. 28.1°C, air temp. 29°C).	50 m TT: 0.8%/0.23 s slower non-significantly (*P* unknown); 0 to 5 m time: 3.2%/0.05 s faster (*P* < 0.05); 15 to 20 m time: 3.9%/0.11 s faster (*P* < 0.05); 5 m split times from 25 to 50 m: no difference; Block time: no difference; Dive distance: no difference; Dive time: no difference; Dive angle at take-off: 12.3% greater (*P* < 0.05); Angle of entry: no difference; Dive velocity: 4.3% faster (*P* = 0.048); Underwater distance: no difference; Stroke rate at 35 m: 0.05 Hz lower (*P* < 0.05); Stroke rate at 15, 20, & 45 m: no difference; Stroke length at 15 m: (% unknown) greater (*P* < 0.05); Stroke length at 20, 35, & 45 m: no difference.
Cuenca-Fernández et al. [37] *‘Usual’ vs ‘PAPE’ conditions only*.	Regional to National *assumed* (*n* = 13, 11 M, 2 F, age M: 18.95 ± 1.63, F: 19.02 ± 0.78).	400 m swim warmup. 8 min transition period. PAPE: 1 set of 4 loaded rep split squats using eccentric flywheel.	Dives.	Average horizontal force: no difference; Average vertical force: no difference; Peak horizontal force: no difference; Peak vertical force: 8.3% greater (*P* = 0.05); Horizontal impulse: no difference; Vertical impulse: 126.6% greater (*P* = 0.04); Resultant impulse: no difference; Horizontal velocity: no difference; Vertical velocity: 169% greater (*P* = 0.05); Resultant velocity: 9.9% greater (*P* = 0.02); Average horizontal velocity: no difference; Average vertical acceleration: 119% greater (*P* = 0.04); Average horizontal power: no difference; Average vertical power: 95.1% greater (*P* = 0.05); Peak horizontal power: no difference; Peak vertical power: 74.6% greater (*P* = 0.04); RFD: 15.9% greater (*P* = 0.04).
Dalamitros et al. [39] *‘Competitive’ group only*.	National (*n* = 11, 11 M, age 20.3 ± 1.8). FINA points = 629.3 ± 78.	1100 m swim warmup. PAPE: 15 min transition phase. CON: 20 min transition phase. PAPE: box jumps onto 40 cm box with 10% of body mass using weighted vest for 5 repetitions of 1 set completed at individualised times (4, 8, or 12 mins) prior to TT.	50 m breaststroke TT from push start.	50 m TT: 0.0%/0 s difference; 0 to 10 m: 1.7%/0.1 s faster non-significantly (*P* = 0.56); 0 to 25 m: 1.8%/0.2 s faster non-significantly (*P* = 0.69); Stroke court: no difference; HR post TT: no difference; tHb post TT: no difference; SmO_2_ post TT: no difference; RPE post TT: no difference.
Waddingham et al. [7] *‘Resistance band squat’* condition.	National (*n* = 10, 8 M, 2 F, age 19 ± 1.25).	830 m swim warmup. 20 min transition period. PAPE: double resistance banded (27 to 68 kg) squats for 3 sets of 3 repetitions with 2 mins between sets completed at 6 mins prior to dive.	Dives.	0 to 15 m: 1.6%/0.11 s faster (*P* = 0.04); CMJ PP: 6.9% greater at 6 mins post.
Waddingham et al. [7] *‘Weighted jump’* condition.	National (*n* = 10, 8 M, 2 F, age 19 ± 1.25).	830 m swim warmup. 20 min transition period. PAPE: CMJ’s with 15% of body mass using weighted vest for 3 sets of 3 repetitions with 2 mins between sets completed 3 mins prior to dive.	Dives.	0 to 15 m: 0.7%/0.05 s slower non-significantly (*P* unknown); CMJ PP: 7.8% greater at 3 mins post.
Waddingham et al. [7] *‘Drop jump’* condition.	National (*n* = 10, 8 M, 2 F, age 19 ± 1.25).	830 m swim warmup. 20 min transition period. PAPE: drop jumps from 45 cm for 2 sets of 5 repetitions with 10 s between repetitions and 3 mins rest between sets 15 s prior to dive.	Dives.	0 to 15 m: 0.4%/0.03 s slower non-significantly (*P* = 1.00); CMJ PP: 2.9% greater at 15 s post.
Ng et al. [43]	Competitive (*n* = 16, 16 M, age 22.1 ± 3.84).	PAPE: 700 m swim warmup. CON: 1400 m swim warmup. 5 min rest. 2 sets of 5 reps of CMJ times 8 min prior.	25 m flutter kick from push start.	Peak thrust: 15.14% greater (*P* = 0.02); Mean thrust: 14.6% greater (*P* = 0.1); Kick speed: 11.6% greater (*P* = 0.01); Kick speed fluctuation: 9.68% lower (*P* = 0.02); Kick frequency: 3.17% greater (*P* = 0.05).
Barbosa et al. [16]	Competitive (*n* = 12, 12 M, age 23.5 ± 3.35).	PAPE: 700 m swim warmup. CON: 1400 m swim warmup. 5 min rest. 2 sets of 5 reps of banded pulls 8 min prior.	25 m front crawl pull from push start.	Peak thrust: 13.37% greater (*P* = 0.01); Mean thrust: 18.9% greater (*P* = 0.05); Thrust-time integral: 18.73% greater (*P* = 0.00); Speed: 2.8%/0.02m/s non-significantly faster (*P* = 0.31); Speed fluctuation: no difference.
Cuenca-Fernández et al. [35] *Pre-training block comparison*.	National (*n* = 14, M: 7, F: 7, age: 18.4 ± 1.41).	400 m swim warmup. 10 min transition phase. PAPE: 1 set 4 loaded reps eccentric flywheel split squats and shoulder extension pull-throughs.	50 m front crawl TT (25 m pool; indoor, water temp. 28.1°C, air temp. 29°C).	50 m TT: 1.3%/0.35 s slower non-significantly (*P* unknown); Dive time: 1.1%/0.01 s slower non-significantly (*P* unknown); 0 to 15 m time: 2.5%/0.18s faster (*P* = 0.028); 0 to 25 m time: 0.4%/0.06 s slower non-significantly (*P* unknown); 0 to 40 m time: 0.9%/0.19 s slower non-significantly (*P* unknown).
Cuenca-Fernández et al. [35] *Post-training block comparison*.	National (*n* = 14, M: 7, F: 7, age: 18.4 ± 1.41).	400 m swim warmup. 10 min transition phase. As above, following a 6 week specific eccentric flywheel PAPE training intervention.	50 m front crawl TT (25 m pool; indoor, water temp. 28.1°C, air temp. 29°C).	50 m TT: 1.2%/0.33 s faster (*P* = 0.024); Dive time: 1.1%/0.01 s slower non-significantly (*P* unknown); 0 to 15 m time: 0.4%/0.03 s faster non-significantly (*P* unknown); 0 to 25 m time: 0.4%/0.06 s faster non-significantly (*P* unknown); 0 to 40 m time: 0.8%/0.17 s faster (*P* = 0.045).
de Arruda et al. [40] *‘Lunge condition’*	National (*n* = 13, M: 13, age: 19.5 ± 3.45). 77% of world-record.	PAPE: 15 min swim warmup. CON: 30 min swim warmup. 10 min transition phase. 1 set of 3 reps of lunges in Smith machine at 85% 1RM completed at individualised times (4, 8, or 12 mins) prior.	50 m front crawl TT (25 m pool; water temp. 27°C).	50 m TT: 0.0%/0 s difference; Dive distance: 2%/7.61 cm greater (ES = 0.3); Flight time: 115.7%/1.03 s greater (ES = 1.06); 0 to 5 m: 11.5%/0.18 s faster (ES = -0.59); 0 to 15 m: 0.1%/0.01 s faster (no difference/trivial); 0 to 25 m: 0.4%/0.05 s faster (no difference/trivial); 25 to 50 m: 1%/0.14 s slower (ES = 0.2); Turn time: 4%/0.13 s faster (ES = -0.39); Stroke frequency 0 to 25 m: no difference/trivial; Stroke frequency 25 to 50 m: no difference/trivial; Stroke length 0 to 25 m: no difference/trivial; Stroke length 25 to 50 m: no difference/trivial; Stroke index 0 to 25 m: no difference/trivial; Stroke index 25 to 50 m: no difference/trivial.
de Arruda et al. [40] *‘Pull-up & box jumps condition’*	National (*n* = 13, M: 13, age: 19.5 ± 3.45). 77% of world-record.	PAPE: 15 min swim warmup. CON: 30 min swim warmup. 10 min transition phase. 1 set of 3RM pull-ups & five 40cm box jumps with 10% of mass completed at individualised times (4, 8, or 12 mins) prior.	50 m front crawl TT (25 m pool; water temp. 27°C).	50 m TT: 1.6%/0.43 s slower (ES = 0.36); Dive distance: no difference/trivial; Flight time: 9%/0.08 greater (ES = 0.39); 0 to 5 m: 7%/0.11 s faster (ES = -0.4); 0 to 15 m: 1.3%/0.1 s slower (ES = 0.24); 0 to 25 m: 1.2%/0.16 s slower (ES = 0.24); 25 to 50 m: 2.1%/0.29 s slower (ES = 0.41); Turn time: 5.5%/0.18 s faster (ES = -0.55); Stroke frequency 0 to 25 m: no difference/trivial; Stroke frequency 25 to 50 m: no difference/trivial; Stroke length 0 to 25 m: no difference/trivial; Stroke length 25 to 50 m: 0.07 m greater (ES = 0.39); Stroke index 0 to 25 m: no difference/trivial; Stroke index 25 to 50 m: no difference/trivial.
de Arruda et al. [40] *‘Pull-up*, *box jump*, *and lunge condition’*	National (*n* = 13, M: 13, age: 19.5 ± 3.45). 77% of world-record.	PAPE: 15 min swim warmup. CON: 30 min swim warmup. 10 min transition phase. 1 set of 3 reps of lunges in Smith machine at 85% 1RM, 3RM pull-ups & five 40cm box jumps with 10% of mass completed at individualised times (4, 8, or 12 mins) prior.	50 m front crawl TT (25 m pool; water temp. 27°C).	50 m TT: 0.4%/0.11 s slower non-significantly (ES = 0.09); Dive distance: 1.7%/6.43 cm greater (ES = 0.23); Flight time: 11.2%/0.1 s greater (**E** = **0**.41); 0 to 5 m: 12.7%/0.2 s faster (ES = -0.77); 0 to 15 m: 0.8%/0.07 s slower non-significantly (no difference/trivial); 0 to 25 m: 0.0% difference; 25 to 50 m: 0.9%/0.12 s slower non-significantly (no difference/trivial); Turn time: 3.9%/0.13 s faster non-significantly (no difference/trivial); Stroke frequency 0 to 25 m: no difference/trivial; Stroke frequency 25 to 50 m: no difference/trivial; Stroke length 0 to 25 m: no difference/trivial; Stroke length 25 to 50 m: no difference/trivial; Stroke index 0 to 25 m: no difference/trivial; Stroke index 25 to 50 m: no difference/trivial.
Cuenca-Fernández et al. [34]	National (*n* = 20, 20 M, age: 18 ± 1.39). FINA points 477 ± 163.	400 m swim warmup. 6 min transition phase. PAPE: 1 set of 3 reps cable shoulder extension pull-through at 85% 1RM.	15 m tethered swim (25 m pool; indoor, water temp. 28.2–28.9°C).	0 to 5 m time: 23.4%/0.99 s slower (*P* = 0.003); Force: 2.6% lower (*P* = 0.001); Acceleration: 30.4% lower (*P* = 0.049); Power: 15% lower (*P* = 0.002); RFD: 10.3% greater (*P* = 0.032); Velocity: 13.7% lower (*P* = 0.001); Stroke rate: 5.1% higher (*P* = 0.044); Stroke length: 19.8% lower (*P* < 0.001); Distance covered: 18% lower (*P* <0.001); Intra-cyclic velocity variation: 17.5% lower (*P* < 0.001).
Crespo et al. [49]	Competitive (n = 17, 10 M, age: 16.6 ± 2, 7 F, age: 15.4 ± 1.8). M FINA points = 402 ± 120, F = 483 ± 102.	580 m swim warmup. 5 min transition phase. PAPE: 1 set of 4 reps of half squats on flywheel.	10 m underwater undulatory swimming.	Time M: 2.2%/0.1 s faster (*P* = 0.01); Time F: 4%/0.3 s faster (*P* = 0.016); Push-off velocity M: 3%/0.8 s faster (*P* = 0.004); Push-off velocity F: 3.7%/0.8 s slower (*P* = 0.14); Average velocity: no difference. Peak velocity: no difference. Average minimum velocity: no difference. Kick frequency: no difference.

Note. CMJ = countermovement jump; F = female; FINA = Fédération Internationale De Natation; HR = heart rate; M = male; PAPE = postactivation performance enhancement; RFD = rate of force development; RM = repetition maximum; RPE = rate of perceived exertion; SmO_2_ = muscle oxygen saturation; TT = time-trial.

### Performance results summary

There was evidence of time-trial or dive improvements reported by at least one study in each of the five protocol categories. There was clear evidence that the shortening of transition phase length improved swimming performance by 1.1–1.5%. Heated clothing garments improved performance by 0.4–0.8%, with a consistent increase of 0.9–2.3°C in skin temperature [4, 19, 21, 41, 45]. Inconsistent outcomes were reported for respiratory interventions with two studies reporting time-trial improvements of 1.1% [47] and 0.9% [44], while another study reported no change [45].

Active warmup improved time-trial outcomes by 0.6–0.9% [4, 21, 33, 44], though with the exception of two studies [4, 44], the physiological mechanisms responsible were not adequately reported. Combination warmup improved performance by 0.8–2% when using heated jackets were used [4, 5, 21]. Combination warmup inclusive of an active dryland warmup and hypoxia led to 3% improvements in 100 m time-trial performance, 0.4°C increases in tympanic temperature, and 10% lower O_2_ saturations [44]. Only two studies [4, 21] reported a relationship between performance and physiological variables. Core temperature pre time-trial demonstrated a positive relationship with performance (R^2^ = 0.91, P < 0.05) when data was pooled across active, passive, and combination protocols [4]. Similarly, higher tympanic temperature pre time-trial was related to performance (R^2^ = -0.647, P = 0.02), however, only during the active dryland protocol with the passive heating and combination protocols reporting no relationship [21].

Upper limb and combined upper and lower limb PAPE protocols demonstrated mostly unfavourable swim performance changes [34, 35, 38, 40, 46]. Lower-limb only PAPE using plyometric actions have demonstrated mixed results [7, 39, 43], though the use of heavier loaded conditions using band resisted squats or loaded lunge and squats have consistently improved time to 15 m [7, 36, 40], dive kinetics [37], and the underwater kick phase [49].

### Study quality

Results for the PEDro scale are illustrated in Table 7. All 25 studies achieved 6–8 points (mean = 7).

**Table 7:** Physiotherapy evidence database (PEDro) scale ratings.

	Q1	Q2	Q3	Q4	Q5	Q6	Q7	Q8	Q9	Q10	Q11	Total
Bagshaw [33]	Yes	Yes	No	Yes	No	No	No	Yes	Yes	Yes	Yes	7
Crespo et al. [49]	Yes	Yes	No	Yes	No	No	No	Yes	Yes	Yes	Yes	7
Cuenca-Fernández et al. [34]	Yes	Yes	No	Yes	No	No	No	Yes	Yes	Yes	Yes	7
Cuenca-Fernández et al. [35]	Yes	Yes	No	Yes	No	No	No	Yes	Yes	Yes	Yes	7
Cuenca-Fernández et al. [36]	Yes	Yes	No	Yes	No	No	No	Yes	Yes	Yes	Yes	7
Cuenca-Fernández et al. [37]	Yes	Yes	No	Yes	No	No	No	Yes	Yes	Yes	Yes	7
Cuenca-Fernández et al. [38]	Yes	Yes	No	Yes	No	No	No	Yes	Yes	Yes	Yes	7
Dalamitros et al. [39]	Yes	Yes	No	Yes	No	No	No	Yes	Yes	Yes	Yes	7
de Arruda et al. [40]	Yes	Yes	No	Yes	No	No	No	No	Yes	Yes	Yes	6
Galbraith and Willmott [41]	Yes	Yes	No	Yes	No	No	No	Yes	Yes	Yes	Yes	7
Knight [21]	Yes	Yes	No	Yes	No	No	No	Yes	Yes	Yes	Yes	7
McGowan et al. [4]	Yes	Yes	No	Yes	No	No	No	Yes	Yes	Yes	Yes	7
McGowan et al. [6]	Yes	Yes	No	Yes	No	No	No	Yes	Yes	Yes	Yes	7
McGowan et al. [5]	Yes	Yes	No	Yes	No	No	No	Yes	Yes	Yes	Yes	7
Neiva et al. [42]	Yes	Yes	No	Yes	No	No	No	Yes	Yes	Yes	Yes	7
Ng et al. [43]	Yes	Yes	No	Yes	No	No	No	Yes	Yes	Yes	Yes	7
Barbosa et al. [16]	Yes	Yes	No	Yes	No	No	No	Yes	Yes	Yes	Yes	7
Ramos-Campo et al. [44]	Yes	Yes	No	Yes	No	No	No	Yes	Yes	Yes	Yes	7
Robertson et al. [45]	Yes	Yes	No	Yes	No	No	No	Yes	Yes	Yes	Yes	7
Sarramian et al. [46]	Yes	Yes	No	Yes	No	No	No	Yes	Yes	Yes	Yes	7
Waddingham et al. [7]	Yes	Yes	No	Yes	No	No	No	Yes	Yes	Yes	Yes	7
West et al. [1]	Yes	Yes	No	Yes	No	No	No	Yes	Yes	Yes	Yes	7
Wilkins and Havenith [19]	Yes	Yes	No	Yes	No	No	No	Yes	Yes	Yes	Yes	7
Wilson et al. [47]	Yes	Yes	No	Yes	Yes	No	No	Yes	Yes	Yes	Yes	8
Zochowski et al. [48]	Yes	Yes	No	Yes	No	No	No	Yes	Yes	Yes	Yes	7

### Risk of bias

The risk of bias analysis is summarised in Table 8. Similar to the assessment of study quality, the categories of allocation concealment and the blinding of participants, administers and assessors frequently caused concern. It should be noted that the possibility of blinding in many of the included studies would not have been possible given the requirement for participants to complete physical activity. This was similar for administers controlling the prescription and timing of these interventions. Blinding assessors of key outcome variables (swimming performance) may have been possible by third-party retrospective analysis or restricting assessor involvement until after the intervention was complete, though this was not clearly described by the included studies.

**Table 8:** Risk of bias.

	Random sequence generation (selection bias)	Allocation concealment (selection bias)	Blinding of participants and personnel (performance bias)	Blinding of outcome assessment (detection bias)	Incomplete outcome data (attrition bias)	Selective reporting (reporting bias)	Other sources of bias (other bias)
Bagshaw [33]	**L**	**S**	**S**	**S**	**L**	**S**	**L**
Crespo et al. [49]	**L**	**S**	**S**	**S**	**L**	**L**	**L**
Cuenca-Fernández et al. [34]	**L**	**S**	**S**	**S**	**L**	**L**	**L**
Cuenca-Fernández et al. [35]	**L**	**S**	**S**	**S**	**L**	**L**	**L**
Cuenca-Fernández et al. [36]	**L**	**S**	**S**	**S**	**L**	**L**	**L**
Cuenca-Fernández et al. [37]	**L**	**S**	**S**	**S**	**L**	**L**	**L**
Cuenca-Fernández et al. [38]	**L**	**S**	**S**	**S**	**L**	**L**	**L**
Dalamitros et al. [39]	**L**	**S**	**S**	**S**	**L**	**L**	**L**
de Arruda et al. [40]	**L**	**S**	**S**	**S**	**L**	**L**	**L**
Galbraith and Willmott [41]	**L**	**S**	**S**	**S**	**L**	**L**	**L**
Knight [21]	**L**	**S**	**S**	**S**	**L**	**L**	**L**
McGowan et al. [4]	**L**	**S**	**S**	**S**	**L**	**L**	**L**
McGowan et al. [6]	**L**	**S**	**S**	**S**	**L**	**L**	**L**
McGowan et al. [5]	**L**	**S**	**S**	**S**	**L**	**L**	**L**
Neiva et al. [42]	**L**	**S**	**S**	**S**	**L**	**L**	**L**
Ng et al. [43]	**L**	**S**	**S**	**S**	**L**	**L**	**L**
Barbosa et al. [16]	**L**	**S**	**S**	**S**	**L**	**L**	**L**
Ramos-Campo et al. [44]	**L**	**S**	**S**	**S**	**L**	**L**	**L**
Robertson et al. [45]	**L**	**S**	**S**	**S**	**L**	**S**	**L**
Sarramian et al. [46]	**L**	**H**	**S**	**S**	**L**	**L**	**L**
Waddingham et al. [7]	**L**	**S**	**S**	**S**	**L**	**L**	**L**
West et al. [1]	**L**	**S**	**S**	**S**	**L**	**L**	**L**
Wilkins and Havenith [19]	**L**	**S**	**S**	**S**	**L**	**L**	**L**
Wilson et al. [47]	**L**	**S**	**S**	**S**	**L**	**L**	**L**
Zochowski et al. [48]	**L**	**S**	**S**	**S**	**L**	**L**	**L**

L indicates low risk, S Indicates some/unclear risk, H indicates high risk.

## Discussion

### Transition phase duration

#### Time-trial performance

Three studies have investigated the effects of altering transition phase length on swimming performance. All three reported an improvement in time-trial performance (*P* < 0.01) during the shorter transition phase with an improvement of 1.5% over 200 m front crawl [1], 1.4% over 200 m main technique in a short-course pool [48], and 1.1% over 100 m front crawl [42]. The variation in split times between the three studies was less consistent with one reporting a 1.5–1.7% improvement from 0 to 150 m in 50 m splits but no difference in final 50 m [1], one reporting a 1.5% improvement 0 to 50 m but only a moderate 0.7% improvement from 50 to 100 m [42], whereas the final study only reported an improvement (% unknown) in the final 100 m [48].

#### Body temperature

Of the two studies [1, 42] that investigated temperature changes only one [1] reported a higher core temperature of 0.3°C between protocols pre time-trial. It is suggested that the difference between studies is largely the result of one study using a 20 vs 45 min transition phase [1] which allowed for a greater core temperature decline compared to smaller 10 mins vs 20 min phase used by the study which reported no change in core or tympanic temperature change pre time-trial between conditions [42].

#### Heart rate

A transition phase of 10 mins demonstrated swimming athletes can sustain a greater HR response following the swimming warmup with an increase of 7 to 15 beats per min during the shorter 10 min transition phase relative to the 20 [42] or 45 min [48] phases. An interesting observation was that both studies that reported a higher initial HR also reported an improvement in the final half of time-trial [42, 48], whereas, the study that did not report a difference in pre time-trial HR did not report a change in the final 50 m of the 200 m time-trial [1]. The impact of this observation is challenging to assess given there is limited evidence to isolate and link elevated initial HR with performance changes.

#### Blood lactate

All three studies investigated La- concentration pre and post time-trial with only one [1] reporting a difference between phase lengths reporting a 2.4 mmol increase following the 20 min protocol compared to the 45 min protocol. It is plausible that this higher La- concentration coincide with elevated $\dot{V}O_{2}$ kinetics which improved the rate of oxygen delivery, preserving muscle function and reducing the rate of fatigue [1, 8].

#### Summary

There is sound evidence that shorter transition phases of 10 to 20 mins prior to 100 to 200 m time-trial’s elicit favourable performance outcomes compared to longer phases, however, from the limited data it is challenging to propose which physiological variables contributed to performance improvements in swimming. It appears that elevated core temperature and HR pre time-trial positively influenced time-trial performance in at least one of these studies. Although it is unlikely the length of transition phases is modifiable in competition, the continuation of these investigations is impactful to determine the key physiological outcomes that should be prioritised when designing transitional phase interventions to acutely enhance swimming performance.

### Passive warmup (passive heating interventions)

#### Time-trial performance

Four studies investigated clothing specific protocols with three using electrically heated jackets at ~50°C [4, 19, 21], while one used non-heated warm clothing consisting of a hooded top and gloves [41]. Changes in performance were varied with a 100 m time-trial improvement of 0.6% [41], whereas practically significant improvements at the elite level of 0.8% in 50 m front-crawl was also were reported by another study [19], as per the smallest worthwhile change in Olympic level swimming being 0.3–0.4% [50, 51]. Additionally, no change in 100 m front-crawl and, although a 0.4%/0.2 s improvement was reported, the practical significance is debatable given the young mean age of participants. Similarly, a marginal improvement of 0.4%/0.3 s in 100 m front-crawl performance was reported with junior swimmers [4], however, the 0.3–0.4% minimum threshold for worthwhile change at the elite level is unable to be applied to this population. Changes in time-trial performance predominantly occurred during the first half of the event following passive heating with improvements frequent in 0 to 15 m [4, 41], 0 to 25 m [19], or split times over the first 50 m [4, 41] reported.

#### Body temperature

Performance enhancements following clothing interventions have chiefly been attributed to alterations in body temperature [8]. Given that increased core [4, 24] or tympanic temperature [21] pre time-trial have demonstrated the ability to enhance time-trial performance up to 100 m, clothing intervention which extend the duration that body temperature can remain elevated following a swimming warmup should lead to consistent positive effects on performance. Of the four studies which included core or tympanic temperature assessment following clothing specific interventions, only one [41] reported a difference between conditions despite similar transition phase durations between studies (20 vs 30 mins). From this, it appears clothing interventions have little effect on core or tympanic temperature once the transition phase exceeds 20 mins. Considering the positive effects on time-trial performance in the absence of core or tympanic temperature change, skin or muscle temperature are likely influential factors.

Each of the studies that reported higher skin temperature pre time-trial [4, 19, 41] reported an improvement in time-trial performance. All three studies [4, 19, 41] reported a change in skin temperature between conditions with increases of 0.87–2.3°C reported, however, it must be noted that skin temperature data are not comparable between research groups due to methodological differences [52]. Despite the possible influence of skin temperature enhancing performance, no study reported the relationship between changes in skin temperature and swimming performance. Two studies [4, 19] reported improvements in performance and increases in skin temperature, yet core or tympanic did not differ from the control condition. To the authors knowledge, only one study has investigated the relationship between swimming performance and pre time-trial skin temperature, concluding the variables were not related [53].

Nevertheless, it is plausible the increase in skin temperature was a contributor to the improved 100 m time-trial performance caused in part by improved dive or free-swim propulsive force [4]. Free-swim metrics including the improvement in stroke efficiency or 25 to 50 m time [4], and the 0.7% in second 50 m performance [41] may reflect this. Further, there is evidence of gender effects as male swimmers reported a 0.6% greater improvement in 50 m time-trial performance, and 16 to 18% greater force and power outcomes during a plyometric push-up compared to female counterparts [19]. It is proposed this gender difference is likely the result of body composition differences between genders with male participants likely having a lower percentage of body fat resulting in higher skin temperatures [54]. In the absence of direct investigation of the relationship between skin temperature increase and swimming performance in these studies, and evidence of the contrary [53], it must be acknowledged these interpretations are observational only. Future research investigating the effects of skin temperature on swimming performance, and the influence of body composition following passive heating are warranted.

#### Thermal perception

Accompanying increases in skin temperature, two studies reported differences in thermal perception [19, 41]. Thermal perception describes both thermal sensation and comfort, both of which were collected via questionnaire scales in all studies that included thermal perception analysis. Thermal sensation scales ask participants to describe how hot/cold they feel, while comfort is used to assess how tolerable that sensation is. One study [19] reported an increase in thermal sensation with participants reporting they felt hotter from 5 mins to the end of the transition phase wearing heated clothing. No change in thermal comfort was reported, though there was a trend for female participants to report they felt hotter after 10 (*P* = 0.056) and 25 mins (*P* = 0.082). Similarly, another study [41] reported a change in thermal comfort with participants reporting they felt hotter wearing the non-heated warmer clothing.

#### Heart rate, blood lactate and RPE

Unlike skin temperature and perception, few changes occurred for HR, La- or RPE. Clothing specific warmup using clothing specific interventions appears to have no effect on HR response pre time-trial [4, 19] or RPE post time-trial [4, 19, 41]. The assessment of La- change is limited with only one study [4] assessing La- post time-trial. The heated clothing condition was the only one to report a change in La- post time-trial compared to the active and combinational conditions. A 1.6 mmol/L increase was reported relative to control. Considering that this condition led to a lower core and skin temperature compared to the active and combination conditions, this La- increase does not appear to be the result of body temperature change.

#### Summary

Currently, it appears that heating using either heated or non-heated warm clothing is a suitable transition phase strategy to enhance swimming performance most likely due to an increase in skin temperature, though investigations (i) correlating skin temperature to performance changes, and (ii) extending the analysis of core temperature are warranted. It appears sprint swimmers would benefit most from this invention as improvements were reported in starts, first 50 m, turns, and second 50 m. More research is required to assess the relationship between body temperature changes and stroke efficiency to establish if swim propulsion is a key variable influencing changes following the start.

### Passive warmup (passive respiratory interventions)

#### Time-trial performance

Three studies investigated the effects of respiratory/hypoxia interventions with protocols including breath holds [45], inspiratory muscle exercise [47], or rest inside a normobaric chamber [44] used. Performance results were mixed with an improvement of 1.1% in 100 m front crawl swum in a short-course pool following two sets of 30 inspirations at 40% of maximal inspiratory muscle pressure, though no physiological changes were reported pre or post time-trial [47]. Another study [44] reported a non-significant 0.9% improvement in 100 m front crawl short-course accompanied by a 6.7% lower SaO_2_ post time-trial. Conversely, no change in any variable was reported by one study [45] which involved three maximal breath holds prior to 400 m front crawl.

#### Physiological effects

In the absence of any cardiovascular variables altering, one group [47] proposed the enhancement in performance following inspiratory muscle exercise may have been caused by an increase in the threshold for activation of the inspiratory muscle metaboreflex [55–57], modification of fatigue perception [58], neural changes [59, 60] or possibly changes in blood flow to the respiratory muscles [47]. For the non-significant 0.9% improvement in 100 m front crawl short-course [44], only SaO_2_ differed between conditions with the rest in hypoxia condition causing a 6.7% reduction. The authors [44] maintain that vasodilation caused by limited O_2_ availability [61] may have increased blood flow and O_2_ delivery which would be influential contributors to performance. Although the only study to involve breath holds failed to report any physiological effects [45], there is substantial room for future research to further investigate these protocols. As raised by the authors [45], the lack of change in haemoglobin (Hb) and splenic volume may indicate the participants did not possess the skill to perform maximal apneas after one familiarisation trial [62], as such, studies with swimmers pre and post a training block specifically focused on maximal apneas development would be of merit to assess the practical use of breath holds to acutely enhance swimming performance.

#### Summary

It appears that interventions which focus on cardiorespiratory protocols can enhance swimming performance from competitive juniors to elite swimmers. While novel strategies are needed to create hypoxic conditions in the competition environment [44], and breath holds require further investigation as skill appears may be a limiting factor [45], the use of inspiratory muscle exercise immediately pre performance has demonstrated a significant change in 100 m front crawl short-course performance [47].

### Active warmup

#### Time-trial performance

Four studies investigated the effects of active dryland warmup during a transition phase with three reporting an improvement of 0.7–0.9% during 100 m front crawl [4, 21], and 0.8% over 200 m main technique [33], whereas one study reported a non-significant 0.7% improvement in 100 m [44]. These improvements appear to be most present in the second half of each time-trial as no study reported an improvement in start performance or split times during the first half of the time-trial. One study [33] reported an improvement in 50 m split time during the third 50 m split which resulted in a 1.8% improvement. This improvement exclusively in the third 50 m split which was significant enough to produce an improvement in overall 200 m performance is of interest being the only study to investigate a distance greater than 100 m [1]. Collectively for the three studies that included start or split times [4, 21, 33], no study reported an improvement during the first half of the time-trial which contrasts the outcomes reported following the heated/warm clothing protocols [4, 19, 41]. This lessens the likelihood of a PAP/PAPE effect having occurred following an active warmup which would likely have occurred early in the TT if at all, and potentially indicates a change in body temperature is a key contributor to start performance and initial free-swim propulsion.

#### Body temperature

Similar to the heated/warm clothing interventions, core or tympanic temperature only differed in one study [44], with three reporting no difference between conditions [4, 21, 33]. However, one study [4] reported a 0.4° C increase in core temperature was trending towards significance (*P* = 0.09). That study [4] was the only to investigate skin temperature failed to change between conditions. As such, it is challenging to include or exclude a change in body temperature following active warmup as a key factor in the favourable time-trial performance outcomes reported, despite evidence that an elevation in core temperature improves swimming performance [4, 24].

#### Heart rate

Similarly, the two studies which investigated HR did not report a difference pre time-trial [4, 33]. One implemented a protocol which increased HR by as much as 29 beats per min which was completed 16 to 21 mins pre time-trial [4], possibly demonstrating this length of time between active warmup completion and pre time-trial was likely too long to maintain an increased HR response. Conversely, another protocol increased HR by ~40 beats per min which was completed 5 mins prior to time-trial [33]. However, it appears that a combination of both a ~20 beats per min decrease occurred during the 5 min rest prior to the time-trial for the active warmup condition, and a ~28 beats per min increase for the control condition over the same time period caused the lack of difference in HR pre time-trial. This rise in HR for the control condition was most likely the result of psychological arousal elevating HR prior to the time-trial [63].

If the elevation of body temperature and HR response was desired, the lack of difference between conditions points to a lack of understanding regarding the required intensity and timing of the activity circuit to create a change immediately pre time-trial. Future research should monitor and report body temperature and HR data throughout various time-points during the transition phase, as opposed to pre and post only. Doing so will allow for a greater understanding of the intensity and timing required to maintain an elevated temperature or HR response above that of homeostasis. Additionally, investigations using swimming populations which compare various dryland active warmup routines focused on creating a body temperature or HR change using limited equipment would be relevant to applied swimming practitioners.

#### Blood lactate and $\dot{V}O_{2}$ kinetics

A change in $\dot{V}O_{2}$ kinetics is a further possible explanation for the changes in performance reported. As the anaerobic energy reserve has a finite capacity, beginning a race with an elevation in $\dot{V}O_{2}$ may allow for the initial sparing of the anaerobic system, allowing for more work to be done anaerobically later in the race [8, 64]. Changes in $\dot{V}O_{2}$ kinetics may also delay the attainment of maximal $\dot{V}O_{2}$ by reducing the $\dot{V}O_{2}$ slow component, or potentially slightly increasing maximal $\dot{V}O_{2}$ [65], both of which may acutely enhance longer distance events. Unlike body temperature [4, 21, 33] and HR [4, 44] which appear to decrease within 20 mins following activity to homeostasis, elevations in $\dot{V}O_{2}$ kinetics can extend to 45 mins post warmup following appropriate exercise intensity [17].

Although the mechanisms responsible for changes in $\dot{V}O_{2}$ kinetics are not well established [64], La- has been used as a proxy variable to assess the magnitude of $\dot{V}O_{2}$ kinetic change [17] with recommendations to begin moderate to high intensity activity of 5–7 mins with a La- of ~3–5 mM [13]. Only one study [4] included La- assessment, as such, conclusions on the effects of acute changes on $\dot{V}O_{2}$ kinetics following dryland intervention cannot be determined currently. Future research should consider frequent La- assessments at the following timepoints; (i) pre and post swim warmup, (ii) pre and post activity circuit, and (iii) pre time-trial to determine the rate at which La- can be maintained following the swim warmup, and/or elevated following the dryland warmup.

#### PAP/PAPE possibility

A PAPE response is also a consideration when active warmup is used. Two studies primarily focused on low intensity lower-limb plyometric actions [21, 33], though a small volume of upper-limb power specific exercises including a plyometric push up [33] and medicine ball throw down were also used [4]. With the exception of one study [44], the three remaining studies [4, 21, 33] did not include a measure pre time-trial to determine if the exercise protocol elicited a PAPE response. Without this, it is challenging to determine if a change, or a lack of change in swim performance was the result of a neuromuscular, thermal, or cardiovascular change. As such, future research investigating active warmup protocols during transition should include a measure of neuromuscular output to better understand the physiology underpinning changes resulting from active warmups. Given that recent research has confirmed the relationship between squat jumps and start performance with elite sprint swimmers [66], changes in pre time-trial jump performance may be impactful for these populations.

#### Summary

Without an evident improvement in body temperature, HR, and a limited assessment of PAPE, the impact on performance is unclear, this may be driven by a physiological outcome that was not investigated. Currently, active warmup protocols during the transition phase appear to have beneficial outcomes for swimming performance, though the factors influencing these improvements are the least understood of the protocols reviewed.

### Combination warmup

#### Time-trial performance

In four studies [4–6, 21], electronically heated clothing garments were worn during the transition phase including during the activity circuit. Three studies reported an improvement of 0.8–2% in 100 m front crawl performance following combination heating compared to control [4, 5, 21], whereas one [6] reported no change during 100 m breaststroke.

Similarly, combination warmup using heated clothing appears to enhance start performance with the same three studies reporting a time-trial improvement also reporting an improvement in 0 to 15 m time of 1.5–3.2% [4, 5, 21]. The key methodological difference between the study which did not report a change in time-trial or start performance [6] relative to the research group’s comparable studies which did [4, 5] was the selection of heated clothing garments used. Unlike the studies which demonstrated changes using heated jackets, the study involving breaststroke swimmers [6] used heated pants during the transition phase. Given breaststroke propulsive force primarily involves the lower limbs [67], the selection of heated pants to specifically isolate an increase in lower limb skin temperature or blood flow is logical. Despite this, comparing the physiological results of this study with the research group’s other two studies [4, 5] which involved near identical transition intervention, core and skin temperature changed the least using heated pants compared to heated jackets which was likely a key contributor to the lack of performance change.

#### Body temperature

An increase in core temperature of 0.13°C pre time-trial [4], and a 0.3°C increase which was trending towards significance (*P* = 0.09) [5] was reported using heating jackets, however the change in core temperature using heated pants was unclear (*P* = 0.36) [6]. Whole body skin temperature when using the heated pants was between 0.16–0.48°C lower compared to using a heated jacket. The likely cause of this difference was the number of heating elements in contact with the skin and their location [6]. Future research should consider and report the number of heating elements and the location of those heating elements in relation to the body when heated clothing garments are used.

#### Heart rate and blood lactate

The activity circuit used by the same three studies [4–6] appeared to have little effect on HR and La- both pre and post time-trial. As previously stated, this is possibly more related to the timing of the intervention than the prescription. Given the relationship between core temperature and performance improvement reported [4], and that the two studies which reported a change in time-trial performance also demonstrated a change in core and skin temperature unlike the breaststroke investigation, it appears from this series of studies that body temperature pre performance is a critical factor affecting swimming performance.

#### PAP/PAPE possibility

Two studies included a CMJ post exercise circuit [5, 6]. Neither study reported a change in CMJ peak power or peak impulse at 8 [6] or 10 mins [5] pre time-trial respectively, yet one [5] reported an improvement in start performance. This result is interested considering the relationship between jump and start performance [66]. Analysis of the relationship between skin or muscle temperature and start performance would be impactful for future research.

#### Summary

One study investigated the effects of an activity circuit completed in hypoxic conditions which led to a 3% improvement in 100 m front crawl [44]. A difference relative to control was reported across several variables with tympanic temperature increasing by 0.4°C, a 10% reduction in arterial O_2_ saturation, and a 1.1% increase in CMJ height all likely influencing the enhancement in time-trial performance. This recent study demonstrates an area transitional phase research should explore further by beginning to combine active warmup with restricted breathing protocols to understand the acute effects of O_2_ alteration with limited physical exertion pre performance.

### Post-activation potentiation (PAP) and performance enhancement (PAPE)

#### Background

Eleven studies have compared the effects of transitional phase PAP/PAPE protocols on swimming performance. PAP has classically described the acute enhancement of muscle function following a maximal or near maximal conditioning activity with the intention of increasing power and/or force during subsequent exercise [14], with recent evidence suggesting this muscle potentiation is chiefly the result of myosin light chain phosphorylation which has a short half-life of ~28 s [31]. Considering numerous publications have included protocols where the stimulus was performed >30 s prior to the subsequent assessment, it has been argued potentiation is not the chief contributor to changes in muscle performance under these conditions [31]. Instead, PAPE has been proposed which includes the combination of muscle temperature, water content and activation. Of the nine studies included in this review, all have latency periods >30 s between the PAP/PAPE stimulus and the beginning of the performance task. Therefore, this review considers all ‘PAP/potentiation’ studies to be defined as PAPE.

Currently, all PAPE research in swimming is focused on sprinting with the longest time-trial distance being 50 m. This focus on sprint performance is likely influenced by work which increased peak vertical and horizontal force production off the diving block following a single set of three repetitions at 87% of 1RM back squats without a swim warmup prior [15]. Following this investigation, swimming research has investigated PAPE conditioning activities using several protocols for the lower limbs [7, 34–37, 39, 40, 43, 46] and also investigated the effects of upper limb protocols [38, 40, 46].

#### Upper-limb PAPE

Three studies have investigated the effectiveness of upper-limb PAPE on swimming performance. Two studies reported decreases of 1.2% in 50 m front crawl [46], and 23.4% in 0 to 5 m performance [34]. Following a set of three pullups at 85% of 1RM, gender effects were seen with males a 1.8% decrease in 50 m performance, whereas no change was seen for the female swimmers [46]. Differences in muscle fibre cross-sectional area and twitch contraction times between genders are possible explanations [68]. Following a set of shoulder extensions at 85% of 1RM [34], an increase in rate of force development and stroke rate was observed during the tethered swim, though multiple variables were negatively affected including; time to 5 m, force, acceleration, impulse, power, and velocity. As acknowledged by the authors [34], fatigue is a plausible explanation for the decrease in performance following PAPE.

Similarly, it is plausible that the decrease in 50 m front crawl performance following the three pullups at 85% of 1RM was mainly the result of fatigue being too great to overcome potentiation [46]. Unlike high intensity PAPE protocols for the lower limb with load recommendations of approximately 80–85% of 1RM, it is plausible this intensity may be too great for the upper limbs given the smaller muscle mass of the upper body [69]. The use of lighter loaded conditions is supported by one study which used resistance banded arm-pulls as the PAPE stimuli at a load of 3–27 kg [16]. Following the PAPE stimulus, peak and mean pulling thrust improved by 13.4 and 18.9%, respectively, during maximal front crawl pull efforts over 25 m. A 2.8% improvement in speed was also reported. Therefore, it currently appears lighter loaded PAPE stimuli is the preferred method of enhancing upper-limb propulsion during front crawl.

#### Combined upper and lower-limb PAPE

Four studies have investigated combined upper and lower limb PAPE protocols, with three reporting no change in 50 m front crawl performance [35, 38, 46] and one [40] reporting a slower 50 m front crawl following a pull-up and box jump protocol. Two studies demonstrated 0 to 5 m time can be enhanced with a PAPE protocol using cable loaded shoulder extensions and bar or flywheel loaded lunges [38], or pull-ups, box jumps, and/or loaded lunges [40]. In contrast to the dive improvements, it appears the negative, or lack of change in 50 m time-trial performance was primarily related to impaired free-swim performance, particularly in the final 25 m [38, 40], though it should be noted the authors [38] did not include a split in the final 25 m and that this assumption is made following statistically faster times through to the 20 m split.

Again, considering one study [40] used heavy pull-ups as the PAPE stimuli for the upper-limbs, it is plausible fatigue was a critical factor which impaired performance after the initial 15 m. However, when combining pull-ups and box jumps with loaded lunges, only a non-significant 0.4% slower time-trial was reported with no change in free-swim splits. The addition of the lunges demonstrated 2.1% faster splits at 15 m compared to the pull-up and box jump only condition, indicating the likely initial velocity increase from the dive following the heavier loaded lunge protocol increased swim velocity mainly through the first lap of the short-course time-trial with led to an overall faster 50 m time-trial relative to the non-lunge protocol.

Considering the lack of time-trial or free-swim improvement following protocols involving heavy pull-ups, the selection of the exercise should be questioned. One group reasoned that the pullup exercise involves joint actions that do not mimic the front crawl stroke action well [46], however, the use of a straight arm pulling exercise [34, 38] is arguably the most specific exercise to mimic the front crawl stroke while retaining the ability for variable loading with a consistent resistance through range. As all studies which used heavy intensities for either the pull-up and shoulder extension exercise failed to enhance free-swimming, this finding reveals that exercise selection may have less influence than intensity in regards to upper-limb PAPE in swimming. Future research should instead focus on comparing varied PAPE intensity prescriptions for the upper-limbs in relation to a control condition without PAPE intervention.

Training experience is also an influential factor when forming PAPE protocols. Only one study [35] investigated the effects of PAPE following a training block specifically focused on exposing participants to the PAPE stimuli used during experimental conditions. Using a split squat and pull-through loaded via an eccentric flywheel, 50 m front crawl in a 25 m pool did not differ between conditions during the initial assessment, though 0 to 15 m performance was improved by 2.5% [35]. Following six weeks of training, 50 m front crawl improved by 1.2%, though no change in 0 to 15 m performance was reported. Accompanying changes in swim performance, an increase in both upper and lower limb strength was seen following the six week block, indicating the change in 50 m front crawl may be the result of a more optimal balance between intensity and fatigue during the free-swim component, though it is difficult to establish why a change in 0 to 15 m performance was not reported.

#### Summary of upper and combined-limb PAPE

Overall, from the studies which have investigated upper and combined limb PAPE protocols it appears that balancing fatigue and potentiation is challenging which has led to unfavourable 50 m front crawl outcomes. This performance decrement is likely the result of muscular fatigue induced by intensity or volume, specifically of the upper-limb PAPE stimuli. Until upper body PAPE is better understood, it is recommended swimming athletes do not attempt to use a PAPE protocol for the upper limbs at loads greater than 3–27 kg [16].

#### Lower-limb PAPE

Eight studies included a condition which was lower limb exclusive [7, 36, 37, 39, 40, 43, 46, 49]. Three studies have investigated the effects of exclusively lower limb PAPE response using a weighted box or countermovement jump protocol [7, 39, 46]. No change in 50 m front crawl [46] or breaststroke [39] performance was reported following ~40 cm box jumps with 10% of body mass added for one set of five repetitions, with no change in 0 to 10 m performance from a push start also reported. Similarly, no change in 0 to 15 m performance was reported following a CMJ protocol with 15% of body mass added, or following a drop jump protocol [7].

When assessing the flutter kick in isolation, a 15.14, 11.6 and 9.7% improvement in peak thrust, kick speed and kick speed fluctuation was seen following unloaded CMJ’s [43]. This is an impactful finding considering the ability for swimmers to utilise plyometric based PAPE protocols has previously been questioned [7]. Considering the upper-body contributes the vast majority of propulsion in front crawl [70], it is likely the studies using unrestricted swimming assessments [39, 46] did not report performance changes as any potential PAPE benefit enhancing the lower limbs would not have been sufficient to facilitate an overall faster 25 or 50 m time-trial. Nevertheless, these results [43] support the use of unloaded CMJ’s the enhance kicking thrust, speed and efficiency.

Heavier loaded protocols were used by five studies [7, 36, 37, 40, 49] with three reporting an improvement in start performance [7, 36, 40], and one study each reporting improvements in dive kinetics [37] or underwater kicking [49]. One study demonstrated improvements in start performance across several time and kinetic related variables up to 15 m with improvements in time to 5 m and 15 m, dive distance, flight time, horizontal velocity, block time, and angular velocity of the knee extensors using an eccentric flywheel with results more favourable compared to a loaded lunge at 85% of 1RM in a Smith machine [36]. In a follow-up investigation, multiple vertical force and velocity variables on the diving block, plus rate of force development were improved following an eccentric flywheel protocol, though horizontal variables remained [37]. Using a 85% of 1RM lunge in a Smith machine, improvements in start performance were reported, though faster splits did not extend beyond the first 5 m, nor did 50 m time-trial performance change [40].

In addition to a loaded CMJ and drop jump protocol, one group [7] also included a band loaded squat which demonstrated an improvement in 0 to 15 m by 1.6%, unlike that of the more plyometric focused protocols included in the study. This difference between heavy and plyometric specific protocols may indicate that swimming athletes respond more favourably to higher intensities compared to power specific plyometric activities for the lower limbs during the start. This lack of improvement following lower limb plyometric or ballistic PAPE is in contrast to studies which have reported performance improvements following these activities [14], though the majority of populations investigated differ from swimmers with regard to their ability to utilise the stretch-shorten cycle as a determinate for success in sports requiring high ground reaction forces. The minimal power output change following the drop jump protocol relative to the CMJ and banded squat protocols highlights skill may have been a limiting factor [7]. Given the lack of need for stretch shorten cycle utilisation in swimming, it is reasonable to assume predominately eccentric derived plyometric protocols is not the optimal method of producing a PAPE response for the lower limbs with most swimmers without targeted intervention in the gym.

#### Summary of lower-limb PAPE

Currently, there is evidence to suggest heavier loaded lower-limb PAPE protocols can enhance swimming performance, specifically during the start component of the race. The key limitation with this research to date has been the validity of the loading stimulus in the competitive environment with most of the research using equipment which cannot be used in competition. Therefore, future research expanding this body of knowledge with equipment which can be used in a call room would be impactful. Additionally, future research comparing volume and intensity prescriptions is warranted.

### Research limitations and recommendations

The vast methodological differences of studies included limits the conclusions and recommendations of this review. This is especially true of the PAPE studies with numerous differences in exercise selection, intensity, and swimming performance assessment used. Similarly, there was limited discussion of results in relation to individual or cohort response. Future assessment of influential factors including gender, anthropometry, body composition, and force generating capacity would all be welcomed, even if sample size were small. Further, an impactful limitation is the lack of accepted average transition phase duration in swimming, evidenced by the numerous different durations used. Except for the requirement that swimmers report to the call room 20 mins pre-event, transition phases fluctuate between competitions and individual swimmer’s routine preferences. Future studies should endeavour to report data at various stages through the transition phase to further the understanding of the impact of time on any variable investigated.

Considering that all studies which did not focus on start performance used a time-trial to assess swim performance, it is problematic that few studies investigated free-swim metrics including splits, velocity, stroke rate, stroke count, or stroke efficiency. Similarly, little emphasis has been placed on defining the race-plan or pacing strategy pre time-trial or describing the athlete’s adherence post time-trial. The reporting of time-trial strategy becomes vital to applied swimming research when attempting to understand at which stage during a time-trial performance is a physiological change occurring. As a result, for most studies which used a time-trial, it is challenging to determine if a change in free-swim performance is predominantly the result of a physiological or pacing change without any information regarding a pre time-trial defined pacing strategy, and at least one free-swim metric. This is magnified when attempting to compare results between studies.

Future research should also consistently report participants mean FINA points or world record ratio to objectively determine the competitive level of athletes to more accurately determine the effect of the intervention in relative to the population investigated. Similarly, more uniformity in the reporting of at least one baseline dryland strength or power measure pre experimental conditions such as a CMJ would allow for a more accurate understanding of the dryland training history and allow practitioners to contextualise the reported outcome to their athlete based on similar physical qualities. This is especially relevant for future PAPE specific investigations.

### Future research directions

There is substantial scope for future research to investigate swim performance following a transition phase. Heated/warm clothing or combination protocols which combine several heated clothing garments is warranted to determine the extent body temperature change has on swim performance. For interventions intended to produce body temperature changes, investigation of a dose-response relationship would be impactful. Future research investigating active or combination interventions should emphasise the reporting of cardiovascular and O2 kinetics response as little is understood regarding a swimmers response to bouts of dryland activity currently. For future PAP/PAPE studies, the practicality of the stimuli needs to be useable in the competition environment. Hence, a greater body of studies using bodyweight and band resisted loaded exercise is warranted. Finally, swimming performance test beyond a time-trial such as repeat sprint tests or step-tests would provide useful information to determine which aspect of swimming a physiological change is impacting which is more challenging when exclusively using time-trial methods of performance assessment.

## Conclusions

There is evidence of transition phase intervention enhancing swimming performance across a range of performance measures, competitive ability, and event distances up to 200 m. Though unlikely to be a modifiable factor, the decrease in transition phase length demonstrates clear improvements in performance, likely as a result of maintaining either body temperature or cardiovascular response following the swim warmup. The use of both heated/warm clothing and combination warmup has demonstrated consistent improvements in starts and overall time-trial performance likely due to the maintenance of core temperature, though the number of heating elements worn appears in clothing specific interventions appears to be an influential factor. Active warmup has demonstrated clear improvements in time-trial performance, though the physiology responsible for the improvements are not well understood, nor has O_2_ kinetic or metabolic changes been sufficiently investigated. PAPE protocols should be used with caution. There is currently limited evidence of time-trial improvement, especially following protocols involving the upper limbs. For the lower limbs, plyometric PAPE of the lower limbs has not benefited time-trial or start performance, though there is evidence of enhanced kick performance. Finally, higher intensity protocols for the lower limbs exclusively appear to have merit in enhancing start performance.

## Supporting information

S1 Checklist PRIMSA check-list. (DOCX)

S1 File Supporting information. (XLSX)

S2 File A4-sized infographic. (TIF)
