# Supplementary figures and images for: Swimming performance, physiology, and post-activation performance enhancement following dryland transition phase warmup: A systematic review
Source: PLoS One. 2022 Aug 18;17(8):e0273248. doi: 10.1371/journal.pone.0273248 (PMC9387820; doi:10.1371/journal.pone.0273248)

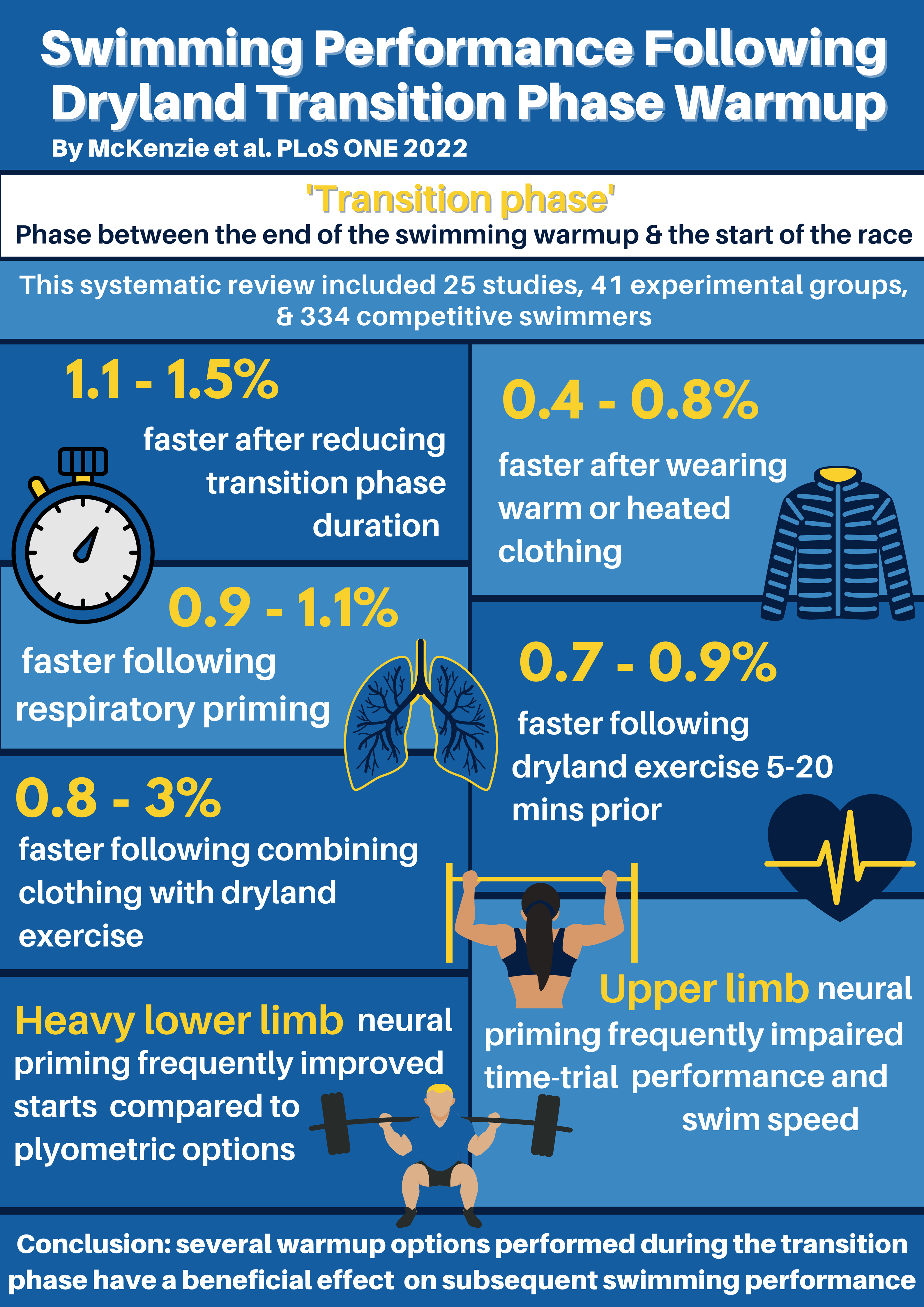

Supplement: S2 File — (TIF) [file pone.0273248.s003.tif]
